# A deep learning model based on self-supervised learning for identifying subtypes of proliferative hepatocellular carcinoma from dynamic contrast-enhanced MRI

**DOI:** 10.1186/s13244-025-01968-w

**Published:** 2025-04-17

**Authors:** Hui Qu, Shuairan Zhang, Xuedan Li, Yuan Miao, Yuxi Han, Ronghui Ju, Xiaoyu Cui, Yiling Li

**Affiliations:** 1 College of Medicine and Biological Information Engineering, Northeastern University, Shenyang, PR China; 2 Department of Gastroenterology, The First Hospital of China Medical University, Shenyang, PR China; 3 Department of Radiology, The First Hospital of China Medical University, Shenyang, PR China; 4 Department of Pathology, The College of Basic Medical Science and the First Hospital of China Medical University, Shenyang, PR China; 5 Department of Radiology, The People’s Hospital of Liaoning Province, Shenyang, PR China

**Keywords:** Hepatocellular carcinoma, Dynamic contrast-enhanced MRI, Deep learning, Self-supervised learning, Prediction model

## Abstract

**Objectives:**

This study employs dynamic contrast-enhanced MRI (DCE-MRI) to noninvasively predict the proliferative subtype of hepatocellular carcinoma (HCC). This subtype is marked by high tumor proliferation and aggressive clinical behavior. We developed a deep learning prediction model that employs a dynamic radiomics workflow and self-supervised learning (SSL). The model analyzes temporal and spatial patterns in DCE-MRI data to identify the proliferative subtype efficiently and accurately. Our goal is to improve diagnostic precision and guide personalized treatment planning.

**Methods:**

This retrospective study included 381 HCC patiephonnts who underwent curative resection at two medical centers. The cohort was divided into the training (*n* = 220), internal (*n* = 93), and external (*n* = 68) test sets. A DL model was developed using DCE-MRI of the primary tumor. Class activation mapping was used to interpret HCC proliferation in HCC.

**Results:**

The pHCC-SSL model performed well in predicting HCC proliferation, with a training set AUC) of 1.00, an internal test set AUC of 0.91, and an external test set AUC of 0.94. Without SSL pre-training, the AUC for internal and external testing decreased to 0.81 and 0.80, respectively. The predictive performance of the derived model was superior to that of the current single-sequence model.

**Conclusions:**

The pHCC-SSL model employs dynamic radiomics and a two-stage training approach to efficiently predict HCC proliferation from multi-sequence DCE-MRI, surpassing traditional single-stage models in accuracy and speed.

**Critical relevance statement:**

Our study introduces the pHCC-SSL model, a self-supervised deep learning approach using DCE-MRI that enhances the diagnostic accuracy of HCC subtypes, significantly advancing clinical radiology by enabling personalized treatment strategies.

**Key Points:**

- The proposed model enables noninvasive identification of HCC with high proliferation and aggressive behavior.
- SSL improves lesion differentiation by reducing redundancy and enhancing feature diversity.
- Dynamic feature extraction captures vascular infiltration, aiding preoperative metastasis risk assessment.

**Graphical Abstract:**

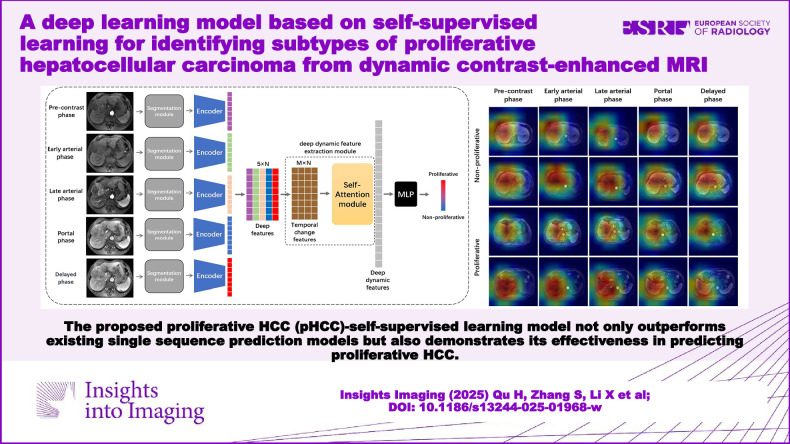

## Introduction

Hepatocellular carcinoma (HCC) originates in the liver and is the third leading cause of cancer-related deaths worldwide [1]. Chronic hepatitis often leads to HCC through stages of fibrosis and cirrhosis [2, 3]. Early-stage HCC tends to be asymptomatic, yet invasive, and is frequently diagnosed late, constraining treatment options and worsening outcomes. Despite advances in surgical and medical therapies, high recurrence rates within two years post-surgery highlight the aggressive nature of these tumors [4]. In particular, rapidly growing, vascularly invasive proliferative HCC indicates a poor prognosis and demands urgent focus [5, 6]. Currently, detection primarily relies on invasive histopathological techniques and presents significant risks and limitations [7].

Magnetic resonance imaging (MRI), particularly dynamic contrast-enhanced MRI (DCE-MRI), plays a crucial role in the noninvasive diagnosis of tumors by detailing vascular structures and assessing invasiveness [8], providing insights into tumor biology through contrast agent dynamics [9]. Although critical for evaluating HCC angiogenesis and improving tumor classification, the full potential of DCE-MRI in HCC diagnosis is underutilized because of the subjective nature of image analysis, which impedes standardized classification and risk assessment [10]. Addressing this subjectivity, especially when diagnosing proliferative HCC, is essential for enhancing the accuracy (ACC) and consistency of DCE-MRI evaluations.

Radiomics harnesses the power of quantitative analysis to unravel the intricacies of tumors from medical images, offering considerable promise for diagnosis and prognostic predictions [11]. The dynamic radiomics approach developed by Qu et al takes this further by extracting richer imaging features from the temporal data in DCE-MRI sequences [12]. This technique improves diagnostic precision and paves the way for precise prediction of HCC progression using multi-temporal DCE-MRI data.

Despite its promise, radiomics faces operational challenges, particularly the time-consuming and labor-intensive manual delineation of regions of interest (ROI) by clinicians, which impedes its integration into routine clinical workflows. This manual process also introduces subjectivity, which affects the consistency and reproducibility of radiomic features [13]. Deep learning (DL) [14, 15], a sophisticated form of artificial intelligence, offers a solution by autonomously analyzing medical images with end-to-end learning models. DL can significantly reduce the burden of manual ROI annotation by identifying complex patterns in imaging data and detecting subtle features that may elude human observation [16–18]. These capabilities enhance the clinical utility of radiomics, potentially transforming cancer care by improving the ACC and personalization of tumor diagnosis and treatment strategies.

This study aims to integrate the concept of dynamic radiomics with DL technologies to explore the application of DL in simulating various dynamic radiomics workflows. Utilizing multiple-sequence data from DCE-MRI, this study focused on developing a model capable of distinguishing between proliferative and non-proliferative HCC phenotypes, with the ultimate goal of enhancing preoperative patient evaluation and tailoring personalized treatment strategies.

## Materials and methods

This study adheres to the principles outlined in the CLEAR (checklist for evaluating radiomics applications) checklist, ensuring transparency and reproducibility in radiomics research. The methodology, including image preprocessing, feature extraction, and model validation, was structured according to radiomics guidelines to enhance rigor and reproducibility [19].

### Study patients

**Fig. 1:**
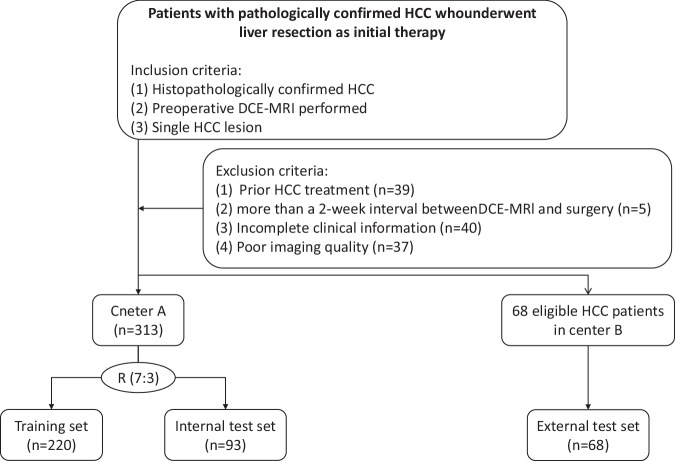
Flowchart of included and excluded patients

This retrospective study was conducted per the Declaration of Helsinki and approved by the Institutional Review Boards of the First Affiliated Hospital of China Medical University (Center A) and Liaoning Provincial People’s Hospital (Center B). Informed consent was waived because of the retrospective nature of this study. Consecutive adult patients (≥ 18 years) who underwent radical resection for HCCs at centers A and B from April 2018 to February 2023 were included. The inclusion criteria were as follows: (1) histopathologically confirmed HCC and (2) preoperative DCE-MRI performed, and a single HCC lesion. The exclusion criteria were as follows: (1) prior treatment for HCC, (2) evidence of extrahepatic metastasis or multiple primary cancers, (3) interval between DCE-MRI and surgery exceeding two weeks, (4) incomplete clinical data, and (5) poor image quality. After applying these criteria, 381 patients with primary HCC were included in the analysis. The detailed selection process is depicted in Fig. 1. All patients in the Center A cohort were randomly divided into training and internal test sets in a 7:3 ratio. In addition, we collected data from 68 patients with HCC from the Center B cohort as an external test set.

### Clinical and radiological features assessment

Clinical features were extracted from patient records, including age, history of HBV and HCV infections, alcohol intake, and AFP levels. Radiological features were assessed from DCE-MRI scans. Key features included tumor size, measured as the maximum tumor diameter on axial images; rim arterial phase hyperenhancement (Rim APHE), characterized by a hyperenhanced rim in the arterial phase of imaging; and delayed central enhancement, identified as areas of hypointensity or delayed enhancement within the tumor in the later phases of DCE-MRI. These radiological features were used to provide further characterization of the tumor and improve model ACC in distinguishing subtypes of proliferative HCC.

### MRI image acquisition

All MR was performed using a 3.0-Tesla (T) MR system (Signa HDxt 3 T, GE Healthcare) with an 8-channel body coil. Non-contrast sequences included a fat-saturated fast spin-echo T2-weighted series (TR/TE: 7500/105 ms) and in-phase/out-of-phase T1-weighted gradient-echo sequences (TR/TE: 260/2.4 ms and 260/3.2 ms). These sequences had a section thickness of 6 mm, an interslice gap of 1.2 mm, and an in-plane resolution of 0.70 mm × 0.93 mm (corresponding to a matrix size of 224 × 320 and a field of view of approximately 160 mm × 300 mm). The total coverage in the axial (*z*) direction was approximately 96 mm, calculated from the number of slices and slice thickness plus the interslice gap.

For DCE-MRI, one precontrast and five postcontrast series were acquired using a fat-suppressed T1-weighted 3D fast-spoiled GE sequence (TR/TE: 3/1.3 ms; flip angle: 15°; section thickness: 5 mm; no gap; matrix: 160 × 276). The in-plane resolution was approximately 1.0 mm × 1.1 mm, with the axial coverage determined by the number of slices and section thickness, covering approximately 100 mm in the *z*-axis. Gadopentetate dimeglumine (Magnevist; Bayer Schering Pharma) was injected intravenously at 0.1 mmol/kg and 2 mL/s, followed by a 20 mL saline flush. The early arterial phase was captured at 15 s, the late arterial phase at 35 s, the portal phase at 35 s, and the delayed phase at 240 s.

### Histopathologic analysis

All specimens from both Center A and Center B were evaluated by the same pathologist with 15 years of experience, who was blinded to both the radiological and clinical data to ensure consistency. The histological classification of HCC was conducted according to the 2019 WHO classification. Immunohistochemical analysis for cytokeratin 19 (CK19) was performed on representative whole tissue sections. HCCs identified as conventional on histological assessment and exhibiting CK19 expression in more than 5% of the tumor cells were classified as CK19-positive conventional HCCs and were categorized within the proliferative subgroup. Additional histological subtypes, including macrotrabecular-massive (MTM), neutrophil-rich, scirrhous, and sarcomatoid HCCs, were similarly categorized as proliferative HCC. Conversely, steatohepatic, clear cell, lymphocyte-rich, and CK19-negative conventional HCCs were classified as non-proliferative HCCs.

### Data preprocessing and selection of the lesion level

The original MRI images were uploaded to the ITK-SNAP medical imaging software (version 3.8.0, available at www.itksnap.org) [20], where the four dynamic enhancement sequences were registered to the pre-contrast sequence. Two attending radiologists with over five years of experience, blinded to both clinical and pathological data, independently selected the slices that best demonstrated the lesions. Any disagreements were resolved through consensus. Five representative lesion slices were obtained from each patient. These slices were then included in the dataset as inputs for the predictive model. In cases where the lesions were too small to provide five distinct slices, all slices containing the lesions were added to the dataset.

### pHCC-SSL

Dynamic radiomics involves four crucial steps: ROI delineation, radiomic feature extraction, dynamic feature construction, and prediction model development. Our pHCC-SSL model adapts to this workflow, creating a two-stage DL architecture comprising pre-training and prediction model training stages. This model seeks to enhance the HCC diagnostic ACC through sophisticated DL computational techniques.

#### Liver segmentation

In radiomics, including dynamic radiomics, precise ROI delineation is critical for feature extraction but is often time-consuming and subjective. In the pHCC-SSL model, radiologists prescreen input images for the presence of tumors, which is essential for accurate HCC detection via MRI. We restricted the input to such images to ensure that the model received only relevant 2D images with lesions, minimizing additional demands on the doctors’ time and effort.

In our multisequence classification model, we implemented a liver segmentation module using the UNet++ network [21], trained on the ATLAS2023 MRI dataset annotated with both liver and tumor regions [22]. By merging these liver and tumor annotations into a unified training dataset for UNet++, this model can accurately segment the entire liver, including both normal liver tissue and tumor regions. This segmentation aids the model in learning features pertinent to the liver and HCC, enhancing interpretability and reducing the reliance on extensive manual input from medical professionals. This approach not only streamlines the workflow but also automates the ROI definition, thereby increasing the automation of the model.

#### Deep feature extraction

**Fig. 2:**
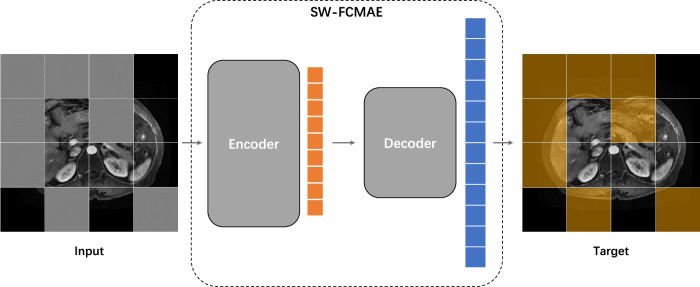
SSL model structure used in pre-training stage

Radiomic features extracted from ROIs using predefined functions lack disease specificity and have limited types, potentially compromising model prediction ACC. In the pHCC-SSL model, we utilized the sample-weighted full-convolution mask autoencoder (SW-FCMAE), a self-supervised learning (SSL) model proposed by Qu et al that trains on patient images without requiring labeled data. As shown in Fig. 2, the SW-FCMAE enhances feature learning by employing a mask autoencoding mechanism and a feature decorrelation module, which diminishes feature redundancy and extracts diverse features better suited for information-rich MRI images. This method allows the encoder to capture the intrinsic characteristics and structures specific to HCC DCE-MRI, thereby improving the relevance and ACC of the model in describing and diagnosing HCC. The specific details of the method are provided in the Supplementary Materials.

**Fig. 3:**
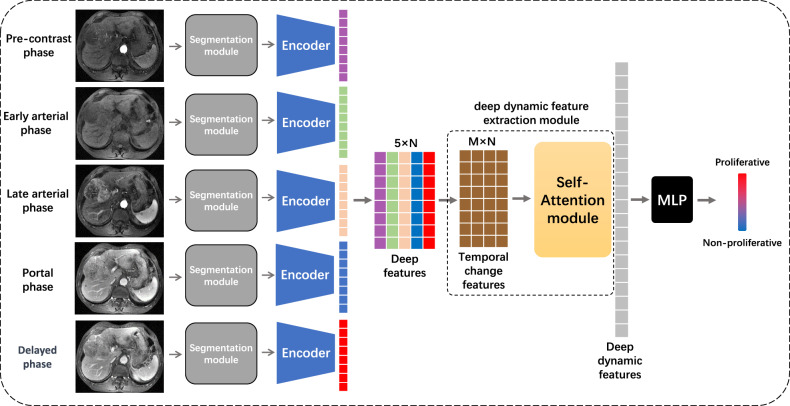
The DL model in the prediction model training stage

During the training stage (Fig. 3), we loaded and fine-tuned the SSL-pretrained encoder to better suit specific prediction tasks. This approach allows the model to utilize complex, unsupervised learned features, enhancing its capability to analyze and predict the DCE-MRI images of patients with HCC.

#### Construction of deep dynamic features

In dynamic radiomics, the construction of dynamic features captures the changing patterns of specific radiomics features over time. In pHCC-SSL, we developed a deep dynamic feature extraction module (Fig. 3) that aggregates identical deep features from various time series into a fully connected layer to extract diverse time-series features indicative of feature changes. These features are then integrated and processed using a self-attention mechanism module.

The self-attention mechanism module applies a multi-head self-attention layer [23]. In this layer, each head computes attention weights using the following formula:

$${{\rm{Attention}}}\left({Q},{K},{V}\right)={{\rm{softmax}}}\left(\frac{{Q}{{K}}^{{{\rm{T}}}}}{\sqrt{{{d}}_{{{\rm{k}}}}}}\right){V}$$

Here, *Q* represents the query matrix, *K* is the key matrix, and *V* is the value matrix derived from the input data. The term *K*^T^ refers to the transposition of the key matrix, where *d*_k_ represents the dimensions of the key vectors used to scale the dot products to ensure stable gradients. The softmax function was applied to convert the scores into probabilities that sum to one.

The outputs from all heads are then concatenated and linearly transformed, allowing the model to focus simultaneously on different parts of the feature sequence. Concatenation merges the outputs from multiple heads and preserves the information captured by each head.

This is followed by a feedforward neural network (FFNN), that processes the attention outputs using the following operation:

$${{\rm{FFNN}}}\left({x}\right)={{\rm{sigma}}}\left({Wx}+{b}\right)$$

where ${x}$ is the input of the FFNN, ${W}$ is the weight matrix, and ${b}$ is the bias vector. The function ${{\rm{sigma}}}$ denotes a nonlinear activation function. In pHCC-SSL, we use a rectified linear unit (ReLU), which introduces nonlinearity into the model, allowing it to learn more complex patterns.

This approach combines multi-head self-attention and an FFNN to simultaneously analyze time-series changes in deep features, enhance data understanding, and generate richer dynamic features.

#### Proliferative prediction of HCC

Dynamic radiomics often uses machine learning algorithms such as support vector machines (SVM) and random forests (RF) to analyze radiomics characteristics and establish prediction models. In pHCC-SSL, we input deep dynamic characteristics into a multilayer perceptron (MLP) [24] to predict the proliferative activity of HCC. This method enables the model to perform gradient backpropagation to support end-to-end learning and model optimization and to ensure prediction accuracy and the model’s effectiveness.

### Training and implementation

We divided the entire training set into training and validation sets in a 7:3 ratio. In the pre-training stage, the SSL model was trained for 100 epochs. We loaded the pre-trained weights from the pre-training stage for the prediction model training stage and trained them for an additional 100 epochs. During training, we utilized binary cross-entropy as the loss function and the Adam optimizer for parameter updates, setting the learning rate to 10^−4^ and the weight decay to 10^−5^. We implemented dynamic data augmentation techniques such as random flipping, rotation, and scaling to enrich the training dataset. The training was conducted over 100 epochs on the PyCharm platform using Python 3.8.16 to fully capture the data features. After training, the performance of the PHCC-SSL model was evaluated using a validation dataset.

### Model visualization

To better understand the decision-making process of the pHCC-SSL model, we employed class activation mapping (CAM) technology [25], which visualizes the model’s focus areas on images, highlighting crucial regions for predictions. We extracted a feature map from each sequence branch’s last convolutional neural network layer and assigned weights to each feature via backpropagation. We generated heat maps by multiplying these weights by the features and applying the ReLU activation function. These heat maps, displayed in red, clearly show the key areas of focus in the model.

### Statistical analysis

Qualitative variables were compared by χ^2^ test or Fisher exact test, and quantitative variables were compared by the Mann–Whitney *U*-test or *t*-test (as appropriate). Receiver operating characteristic (ROC) curves were used to evaluate the probability of correctly evaluating the proliferation in the training, verification, and test queues. The area under the curve (AUC) with a 95% confidence interval, ACC, sensitivity, specificity, positive predictive rate (PPV), and negative predictive rate (NPV) were calculated. Python (v3 8.16, https://www.python.org/) was used for the statistical analysis. All tests were bilateral, and *p* < 0.05 was considered statistically significant.

## Results

### Baseline characteristics

This study included a cohort of 353 patients with HCC divided into a training group of 220 patients, an internal test group of 93 patients, and an external test group of 68 patients. As shown in Table 1, non-proliferative HCC was more common than proliferative HCC in all evaluation groups (training group: 24.1% proliferative HCC, 53/220; internal test group: 23.7% proliferative HCC, 22/93; external test group: 22.0% proliferative HCC, 15/68). Statistically, there was no significant difference in the distribution of all characteristics among the three groups; the *p* values for all characteristics were greater than 0.05.

**Table 1:** Baseline characteristics of study sets

Characteristics	Training set (*n* = 220)	Internal test set (*n* = 93)	External test set (*n* = 68)	*p* value
Subtype				0.705
Conventional positive	42 (19.1)	13 (14.0)	11 (16.3)	
MTM	10 (4.5)	7 (7.5)	3 (4.4)	
Sarcomatoid	1 (0.5)	2 (2.2)	0 (0.0)	
Conventional negative	153 (69.5)	62 (66.7)	50 (73.5)	
Clear cell	7 (3.2)	6 (6.5)	2 (2.9)	
Lymphocyte-rich	2 (0.9)	2 (2.2)	0 (0.0)	
Steatohepatitis	5 (2.3)	1 (1.1)	2 (2.9)	
Age (years)	58.3 (8.9)	58.7 (10.1)	58.6 (± 7.0)	0.943
Sex				0.823
Female	32 (14.5)	16 (17.2)	10 (14.7)	
Male	188 (85.5)	77 (82.8)	58 (85.3)	
HBV				0.052
Negative	109 (49.5)	56 (60.2)	45 (66.2)	
Positive	111 (50.5)	37 (39.8)	23 (33.8)	
HCV				0.971
Negative	191 (86.8)	80 (86.0)	58 (85.3)	
Positive	29 (13.2)	13 (14.0)	10 (14.7)	
Alcohol consumption				
Negative	174 (79.1)	75 (80.6)	50 (73.5)	0.661
Positive	46 (20.9)	18 (19.4)	18 (26.5)	
AFP (ng/mL)	311.4 (388.6)	375.5 (441.3)	290.5 (362.3)	0.363
Tumor size (cm)	4.5 (1.4)	4.9 (1.4)	4.8 (1.2)	0.114
Rim APHE				0.726
Negative	131 (59.5)	53 (57.0)	44 (64.7)	
Positive	89 (40.5)	40 (43.0)	24 (35.3)	
Proliferative				0.933
Non-proliferative	167 (75.9)	71 (76.3)	53 (78.0)	
Proliferative	53 (24.1)	22 (23.7)	15 (22.0)	

*MTM* macrotrabecular-massive, *HBV* hepatitis B virus, *HCV* hepatitis C virus, *AFP* alpha-fetoprotein, *Rim APHE* arterial phase hyperenhancement

Unless otherwise stated, categorical variables are presented as a number of patients (percentage); continuous variables are presented as the mean (standard deviation)

*p* value was calculated by the Chi-square test or the *t*-test

### Model performance of pHCC-SSL

**Fig. 4:**
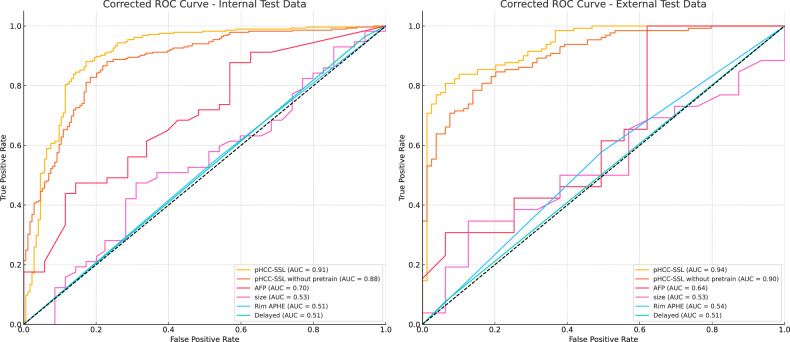
The ROC curves of the pHCC-SSL model, the classification model trained directly without the pre-training stage, and several clinical and radiological indicators, shown in the internal test set (left) and external test set (right)

The pHCC-SSL model achieved perfect scores (ACC, AUC, sensitivity, specificity, NPV, and PPV of 1.00) for the training set, confirming model convergence. The scores in the internal test set were 0.86, 0.91, 0.83, 0.87, 0.89, and 0.80, respectively; in the external test set, they were 0.84, 0.94, 0.80, 0.87, 0.88, and 0.79, respectively. Ablation experiments compared pHCC-SSL with a model trained without an SSL-based pre-training stage. As shown in Table 2 and Fig. 4, the latter model, despite converging with perfect scores in training, performed lower in the test sets: the internal test set scores were ACC 0.81, AUC 0.88, sensitivity 0.83, specificity 0.81, NPV 0.88, and PPV 0.72; and the external test set scores were ACC 0.80, AUC 0.90, sensitivity 0.86, specificity 0.76, NPV 0.90, and PPV 0.69. This illustrates that the SSL pre-training stage significantly enhanced the model’s ability to analyze the DCE-MRI images of patients with HCC and improved its predictive performance.

**Table 2:** Performance of the pHCC-SSL and pHCC-SSL without SSL pretrain in the training and test sets

	ACC	AUC (95% CI)	Sensitivity	Specificity	NPV	PPV
pHCC-SSL						
Training set	1.00	1.00 (1.00–1.00)	1.00	1.00	1.00	1.00
Internal test set	0.86	0.91 (0.87–0.93)	0.83	0.87	0.89	0.80
External test set	0.84	0.94 (0.90–0.97)	0.80	0.87	0.88	0.79
pHCC-SSL without pretrain						
Training set	1.00	1.00 (1.00–1.00)	1.00	1.00	1.00	1.00
Internal test set	0.81	0.88 (0.84–0.91)	0.83	0.81	0.88	0.72
External test set	0.80	0.90 (0.86–0.94)	0.86	0.76	0.90	0.69

We compared it to five single-sequence prediction models to demonstrate that pHCC-SSL can extract more informative features from multi-sequence images. All models, including single-sequence models, utilized the same encoder architecture and were equipped with SSL pre-trained weights to neutralize the effects of SSL pre-training on their predictive capabilities. As shown in Table 3, pHCC-SSL outperformed the other methods regarding both ACC and AUC on the internal and external test datasets.

**Table 3:** Performance of the pHCC-SSL and five single-sequence prediction models in the training and test sets

	Training set		Internal test set		External test set	
	ACC (%)	AUC (95% CI)	ACC (%)	AUC (95% CI)	ACC (%)	AUC (95% CI)
pHCC-SSL	1.00	1.00 (1.00–1.00)	0.86	0.91 (0.87–0.93)	0.84	0.94 (0.90–0.97)
Pre-contrast	0.98	1.00 (0.99–1.00)	0.82	0.83 (0.79–0.87)	0.80	0.87 (0.82–0.92)
Early arterial	0.98	1.00 (1.00–1.00)	0.81	0.84 (0.80–0.88)	0.83	0.91 (0.87–0.95)
Late arterial	0.98	1.00 (1.00–1.00)	0.82	0.84 (0.80–0.88)	0.82	0.91 (0.86–0.94)
Portal	0.97	1.00 (0.99–1.00)	0.82	0.85 (0.81–0.89)	0.78	0.87 (0.82–0.92)
Delayed	0.98	1.00 (1.00–1.00)	0.82	0.84 (0.80–0.88)	0.80	0.88 (0.84–0.93)

In addition to traditional clinical-radiological features, we selected tumor size, rim APHE, and delayed central enhancement based on previous studies identifying their association with HCC proliferative potential [26, 27]. These radiological features, along with the tumor marker alpha-fetoprotein (AFP), were included in both univariate and multivariate analyses of the training set (Table 4). Although both AFP and the pHCC-SSL score demonstrated statistical significance in multivariate analysis, their predictive performances varied significantly, as evidenced by the ROC curves in the internal and external test sets. Specifically, the pHCC-SSL score exhibited superior predictive performance, with an AUC of 0.91 in the internal test set and 0.94 in the external test set, compared to AFP’s AUC of 0.70 and 0.64, respectively (Fig. 4). These findings highlight the substantial incremental value of radiomics features over traditional clinical-radiological features and tumor markers like AFP in predicting the proliferative behavior of HCC.

**Table 4:** Predictors for identifying proliferative HCCs at logistic regression analysis in the training set

	Univariable			Multivariable		
	OR	95% CI	*p*	OR	95% CI	*p*
Tumor size	1.03	0.87–1.22	0.742	–	–	–
Rim APHE	1.17	0.73–1.86	0.512	–	–	–
Delayed central enhancement	1.49	0.59–3.79	0.4	–	–	–
AFP	11.37	4.99–25.9	< 0.001	7.65	2.13–27.45	0.002
pHCC-SSL score	156.14	62.35–391.02	< 0.001	142.71	55.1–369.61	< 0.001

### Model visualization

**Fig. 5:**
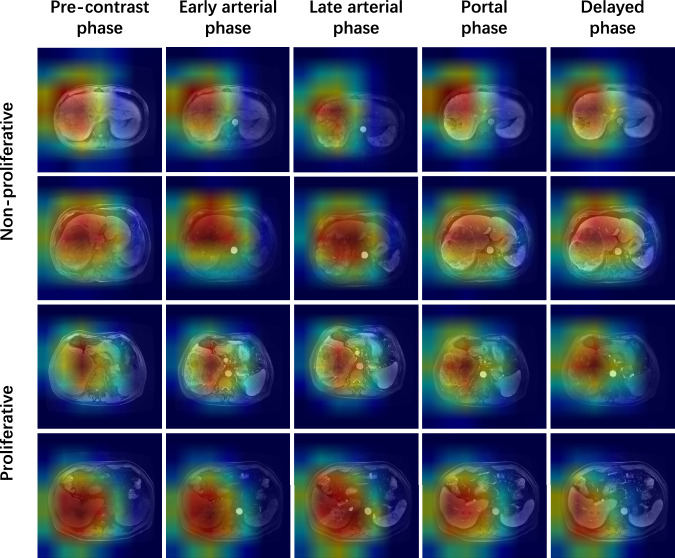
Visualization of four patient examples. Patients in the first and second rows were diagnosed with non-proliferative HCC, while patients in the third and fourth rows were diagnosed with proliferative HCC. The first to fifth columns display the CAM calculated based on the sequence images of the pre-contrast, early arterial, late arterial, portal, and delay phases, respectively

The CAM heat map showed that the active regions of the network in the five MRI sequences were all in tumor lesions (Fig. 5), but there were differences among the different sequences. Some sequences focus on the tumor parenchyma, whereas others focus on the junction between the tumor and normal liver tissue. In proliferative HCC patients, the heat map from pHCC-SSL tended to be highlighted in the tumor area, whereas in non-proliferative HCC patients, the heat map area was scattered.

## Discussion

In this study, we developed a DL model tailored for multi-sequence DCE-MRI data to predict HCC proliferation using a two-stage training method. This approach effectively extracts deep features from five MRI sequences and generates dynamic deep features that reflect temporal changes using a deep dynamic feature extraction module. The model’s performance in predicting HCC proliferation exceeded that of both the single-stage training and traditional single-sequence models.

Proliferative HCC is a highly aggressive subtype, and early and accurate diagnosis is crucial for effective treatment [28]. While liver biopsy has been the traditional diagnostic method, its invasive nature and associated risks highlight the need for noninvasive alternatives. Advanced imaging techniques, such as multiparametric MRI, offer a safer, noninvasive approach that enables earlier detection and more frequent monitoring of tumor characteristics, allowing for personalized and timely therapeutic interventions. Conventional DCE-MRI features, such as rim arterial phase hyperenhancement (Rim APHE) and tumor shape, are valuable, but the complexity of proliferative HCC may be overlooked [27, 29, 30]. In contrast, radiomics and DL models such as pHCC-SSL analyze intricate imaging data and reveal subtle tumor characteristics that enhance diagnostic ACC. This advancement in diagnostic technology not only improves the classification of HCC subtypes but also directly impacts treatment decisions, leading to more precise and effective therapies and, ultimately, better patient outcomes [26, 27].

The pHCC-SSL model differentiates itself from other multi-sequence image prediction models by utilizing SSL to pretrain its encoder, thus enhancing its capability to extract structural and detailed features from DCE-MRI images [31, 32]. This strategy has significantly improved the model’s interpretability of complex biomedical images. In addition, inspired by dynamic radiomics, we introduced a method for constructing dynamic deep features that recognize time-series variation rules across different sequences [33] using a self-attention module to transform these into dynamic depth features. This method enhances the model’s ACC in analyzing time-series data and opens new research avenues for medical image processing. To aid clinical applications and enhance interpretability, we developed a thermographic tool using CAM technology, enabling quick and intuitive diagnostic support by visually highlighting tumor proliferation. This non-invasive approach simplifies the diagnostic process and aids clinicians in devising tailored treatment plans for proliferative HCC, thereby improving the efficiency and ACC of medical decision-making.

However, this study has several limitations. First, due to the study’s retrospective design, selection bias was inevitably introduced. Second, the study was mainly conducted in areas where hepatitis B virus (HBV) was prevalent, which may limit the model’s universality to areas with diverse etiologies. Additionally, although histopathology is considered the gold standard for diagnosing proliferative HCC, intratumoral heterogeneity and the localized nature of tissue sampling can result in misclassification. At the same time, for the construction of the DL model, the amount of data in this study was relatively small, which needs to be improved by expanding the sample size in the future, including retrospective or prospective research at more centers. In addition, the difference in the number of patients with proliferative and non-proliferative liver cancer may also affect the model’s generalization ability, resulting in a large difference between the model’s performance on the training and test sets. Moreover, the model exhibited signs of overfitting, as indicated by the perfect performance on the training set compared to the slightly lower AUCs on the test sets. To address this issue, future studies should focus on increasing sample size, incorporating more diverse patient populations, and optimizing model training techniques such as data augmentation, dropout, and more extensive external validation. These efforts will help improve the robustness and clinical applicability of the proposed model.

## Conclusion

In this study, we proposed pHCC-SSL, a DL model for DCE-MRI. It efficiently extracts deep features from each sequence and generates deep dynamic features that reflect time-series changes. The model exhibited satisfactory performance in a multicenter cohort test and effectively identified proliferative HCC. This achievement provides a new direction for noninvasive and efficient prediction of HCC in the future. It shows the great potential of DL technology in the field of medical image analysis.

## Supplementary information

ELECTRONIC SUPPLEMENTARY MATERIAL

## Data Availability

Due to privacy restrictions, the datasets presented in this article are not publicly available. Requests to access the datasets should be directed to Yiling Li, lyl-72@163.com. Please refer to the Supplementary Materials for the relevant codes of this study.

## Abbreviations

ACC: Accuracy
AUC: Area under the curve
CAM: Class activation mapping
CK19: Cytokeratin 19
DCE-MRI: Dynamic contrast-enhanced magnetic resonance imaging
DL: Deep learning
FFNN: Feedforward neural network
HBV: Hepatitis B virus
HCC: Hepatocellular carcinoma
MRI: Magnetic resonance imaging
MTM: Macrotrabecular-massive
NPV: Negative predictive rate
PPV: Positive predictive rate
ReLU: Rectified linear unit
ROC: Receiver operating characteristic
ROI: Regions of interest
SSL: Self-supervised learning
SW-FCMAE: Sample-weighted full-convolution mask autoencoder
