# Supplementary material for: A deep learning model based on self-supervised learning for identifying subtypes of proliferative hepatocellular carcinoma from dynamic contrast-enhanced MRI
Source: Insights Imaging. 2025 Apr 17;16:89. doi: 10.1186/s13244-025-01968-w (PMC12006648; doi:10.1186/s13244-025-01968-w)
Supplement: Supplementary file 1 — ELECTRONIC SUPPLEMENTARY MATERIAL [file 13244_2025_1968_MOESM1_ESM.pdf]

A deep learning model based on self-supervised learning for identifying subtypes of proliferative hepatocellular carcinoma from dynamic contrast-enhanced MRI

ELECTRONIC SUPPLEMENTARY MATERIAL

Implementation details of deep feature extraction

During the pre-training phase, the SW-FCMAE follows the following formulas:

$$\text{Encoder: } \mathrm{h} = \mathrm{f}_{\theta}\big(\mathrm{x} \odot (1 - \mathrm{m})\big)$$

$$\text{Decoder: } \hat{\mathrm{x}} = \mathrm{g}_{\phi}(\mathrm{h})$$

Here, x is the original input image, m is the mask applied to the image, h is the hidden representation generated by the encoder f_{θ}, and $\hat{\mathrm{x}}$ is the image reconstructed by the decoder g_{ϕ}. The encoder is responsible for mapping the unmasked parts of the input image to a latent space, whereas the decoder attempts to reconstruct the entire image, including the masked portions.

The model was trained by minimizing the difference between the input image x and reconstructed image $\hat{\mathrm{x}}$, that is, by minimizing the following loss function:

$$L_{\text{reconstruction}} = \| \mathrm{x} - \hat{\mathrm{x}} \|^2$$

Additionally, the SW-FCMAE includes a feature-decorrelation module to reduce the correlation among features and enhance feature independence. This module adjusts the loss function using regularization terms based on Random Fourier Features (RFF).

The mapping into the RFF space is defined as:

$$\phi(x) = \sqrt{\frac{2}{D}} \cos(Wx + b)$$

where, W is a random Gaussian matrix, b is a random vector, and D is the dimensionality of the RFF space.

The correlation matrix C is computed as:

$$C = \frac{1}{n} \sum_{i=1}^{n} (w_i x_i - \mu)\,(w_i x_i - \mu)^T$$

where w_i is the weight of the i-th feature, x_i is the feature vector, and μ is the mean feature vector.

The overall loss function L is:

$$L = L_{\text{reconstruction}} + \lambda \sum_{i \neq j} C_{ij}^2$$

where $L_{\text{reconstruction}}$ is the reconstruction loss described above, λ is a regularization parameter, and C_{ij}represents the off-diagonal elements of the correlation matrix C. This encourages the model to capture more diversified features by minimizing correlations.
